# Antibiotics in Bone Cements Used for Prosthesis Fixation: An Efficient Way to Prevent *Staphylococcus aureus* and *Staphylococcus epidermidis* Prosthetic Joint Infection

**DOI:** 10.3389/fmed.2020.576231

**Published:** 2021-01-20

**Authors:** Andréa Cara, Mathilde Ballet, Claire Hemery, Tristan Ferry, Frédéric Laurent, Jérôme Josse

**Affiliations:** ^1^Centre International de Recherche en Infectiologie, CIRI, Inserm U1111, CNRS UMR5308, ENS de Lyon, UCBL1, Lyon, France; ^2^Laboratoire de Bactériologie, Institut des Agents Infectieux, Hôpital de la Croix-Rousse, Hospices Civils de Lyon, Lyon, France; ^3^Université Claude Bernard Lyon 1, Lyon, France; ^4^Service de Maladies Infectieuses, Hôpital de la Croix-Rousse, Hospices Civils de Lyon, Lyon, France; ^5^Centre Interrégional de Référence des Infections Ostéo-Articulaires complexes (CRIOAc Lyon), Hospices Civils de Lyon, Lyon, France

**Keywords:** arthroplasty, prosthetic joint infection, biofilm, *Staphylococcus*, antibiotic loaded bone cement

## Abstract

Prosthetic joint infections (PJIs) are one of the most frequent reasons for arthroplasty revision. These infections are mostly associated with the formation of biofilm, notably by staphylococci, such as *Staphylococcus aureus* and *Staphylococcus epidermidis*. To minimize the rates of PJIs following primary or revision total joint arthroplasty, antibiotic-loaded bone cements (ALBCs) can be used for prosthesis fixation. However, its use is still debated. Indeed, various studies reported opposite results. In this context, we aimed to compare the prophylactic anti-biofilm activity of ALBCs loaded with two antibiotics with ALBC loaded with only one antibiotic. We compared commercial ready-to-use cements containing gentamicin alone, gentamicin plus vancomycin, and gentamicin plus clindamycin to plain cement (no antibiotic), investigating staphylococcal biofilm formation for 10 strains of *S. aureus* and *S. epidermidis* with specific resistance to gentamicin, vancomycin, or clindamycin. Firstly, we performed disk diffusion assays with the elution solutions. We reported that only the cement containing gentamicin and clindamycin was able to inhibit bacterial growth at Day 9, whereas cements with gentamicin only or gentamicin and vancomycin lost their antibacterial activity at Day 3. Then, we observed that all the tested ALBCs can inhibit biofilm formation by methicillin-susceptible staphylococci without other antibiotic resistance ability. Similar results were observed when we tested vancomycin-resistant or clindamycin-resistant staphylococci, with some strain-dependent significant increase of efficacy for the two antibiotic ALBCs when compared with gentamicin-loaded cement. However, adding vancomycin or clindamycin to gentamicin allows a better inhibition of biofilm formation when gentamicin-resistant strains were used. Our *in vitro* results suggest that using commercially available bone cements loaded with gentamicin plus vancomycin or clindamycin for prosthesis fixation can help in preventing staphylococcal PJIs following primary arthroplasties, non-septic revisions or septic revisions, especially to prevent PJIs caused by gentamicin-resistant staphylococci.

## Introduction

The number of primary and revision total joint arthroplasty (TJA) has risen over the last decades. In the US, primary total hip arthroplasties (THAs) and total knee arthroplasties (TKAs) are projected to reach 635,000 and 935,000 procedures, respectively, in 2030 (1). With aseptic loosening, infection is a major cause for arthroplasty revision, especially in early failures after TKA (2). In a recent French study on a cohort of 1,170 reinterventions after TKA, prosthetic joint infection (PJI) accounts for almost 50% of total revision (3). PJIs occur after 1–7% of TJA (4). The most incriminated pathogens are staphylococci, especially *Staphylococcus aureus*, mostly in early and delayed PJIs, and *Staphylococcus epidermidis*, mostly in late chronic or exacerbated PJIs (5, 6). Staphylococcal PJIs can be complicated to treat, and it is partially due to the ability of staphylococci to form biofilm. Biofilms are communities of bacteria embedded in an extracellular matrix. The formation of biofilm is classically described in three main phases: (i) initial attachment, (ii) production of extracellular matrix and cell proliferation, and (iii) biofilm structuring and cell detachment (7). The first phase, initial attachment, is critical for biofilm formation. Indeed, when staphylococci start to produce their extracellular matrix and structure as biofilm, it confers to bacteria some properties of tolerance against antibiotics (8). Indeed, a subpopulation of bacteria inside biofilms faces a lack of nutrients and oxygen. These conditions lead to a decrease of metabolic activity and an increase of antibiotic tolerance, explaining the difficulty to treat biofilm-associated infections (9). Biofilms were reported to be tolerant to antibiotic concentrations 10–1,000-fold superior to the minimal inhibition concentrations (MICs) determined for planktonic bacteria (10).

Preventing the adherence of planktonic bacteria to the prosthesis or the early steps of the formation of other biofilm-like structures that happen in the first hours or days after the prosthesis implantation is a key point to fight PJIs. During this early time, the race to the surface took place. Tissue cells have to colonize the implant before the bacteria to permit the prosthesis integration and prevent bacteria to form biofilm (11). To prevent PJIs following primary or revision TJA, antibiotic-loaded bone cements (ALBC) can be used for prosthesis fixation. Bone cements are composed of polymethyl methacrylate (PMMA). Initially, PMMA can only polymerize at high temperatures, so it cannot be used for medical applications. But, a new method for polymerizing PMMA at room temperature was developed in 1943, allowing its use for prosthesis fixation (12). In prophylactic situations, bone cements can be loaded with low doses of antibiotics (between 0.5 and 2 g antibiotics/40 g PMMA) to prevent PJIs with a limited impact on the mechanical properties of cement.

Prophylactic ALBCs are commonly used in Europe, especially in Scandinavian countries. It was mostly justified by previous studies based on the Norwegian arthroplasty register showing that systemic antibiotics combined with ALBCs for prosthesis fixation led to fewer revisions than systemic antibiotics or ALBC alone following THA (13). Moreover, another study from 2006 reported that the risk of THA revision due to PJI was equivalent for uncemented and for cemented arthroplasties with ALBC, but higher for cemented arthroplasties without antibiotic cement (14). Similar results were published in a recent meta-analysis about implant fixation and the risk of PJI in THA (15). The authors reported that all cemented prostheses (cemented fixations, hybrid fixations, reverse hybrid fixations) were each associated with an increase of PJI risk when compared with uncemented prosthesis. For ALBCs, the risk of PJI was reduced when compared with cemented fixations. When ALBCs were compared with uncemented fixations, the authors did not report any difference concerning the PJI risk. However, the same group performed a meta-analysis about implant fixation and the risk of infection in TKA. Their observation suggests that uncemented fixation may be associated with lower PJI risk in primary TKA than cemented fixation, and that the use of ALBC may be associated with increased PJI risk when compared with plain cement (16). To note, Sultan et al. highlighted that the question about the use of ALBCs in TJA is more relevant in TKA than in THA as most of the patients received cemented implants in TKA, whereas cementless prostheses were more and more used in THA (4). They also highlighted a potential bias regarding patient selection, suggesting that patients with high risk of infection (diabetes mellitus, obesity) were more subject to TJA with ALBCs.

However, the type of ALBCs (handmade or ready-to-use, which antibiotic(s) is/are loaded) is rarely questioned in these studies about primary TJA. Moreover, the choice of the antibiotics can largely influence the development of PJIs. Indeed, gentamicin is mostly used alone in ALBCs in primary TJA, and the percentage of gentamicin resistance is important in staphylococci. In 1999, Schmitz et al. investigated the prevalence of gentamicin resistance in staphylococci in 19 different European hospitals. Of the *S. aureus* isolates, 23% were resistant to gentamicin. They reported that resistance to gentamicin is more frequent in methicillin-resistant *Staphylococcus aureus* (MRSA) isolates (75%) than in methicillin-susceptible *Staphylococcus aureus* (MSSA) isolates (4%). Of the coagulase-negative staphylococci (CNS) isolates, 33% of the strains were reported to be resistant to gentamicin. For methicillin-resistant CNS (MRCNS) isolates, the prevalence of gentamicin resistance was 48%, whereas it was 7% for methicillin-susceptible CNS (MSCNS) (17). However, the strains were isolated from blood, hospital-acquired pneumonia, or skin and soft tissue infections, not from PJIs. In 2009, Hellmark et al. reported that, in a collection of 33 *S. epidermidis* isolated during revision surgery for PJI in two Swedish hospitals, 84% of the isolates were resistant to oxacillin, 79% were resistant to gentamicin, and 67% were resistant to clindamycin, whereas no isolate was reported to be resistant to vancomycin (18). The authors also suggested that the high gentamicin resistance could be related to the common use of gentamicin-loaded cement on previous surgeries.

In this context, we aim to investigate the prophylactic anti-biofilm activity of ALBCs with two antibiotics destined to prosthesis fixation. We compared commercial ready-to-use ALBCs containing gentamicin alone, gentamicin plus vancomycin, and gentamicin plus clindamycin to plain cement (no antibiotic), investigating staphylococcal biofilm formation in elution solutions from these four cements.

## Materials and Methods

### Bacterial Strains

A collection of 10 strains of *S. aureus* and *S. epidermidis* was used in this study. We used the MSSA SH1000, a reference strain routinely used in our laboratory for biofilm experiments (considered as our MSSA strain) and nine clinical strains. These methicillin-susceptible or -resistant clinical strains were isolated during routine work performed at the Bacteriology Department of Hôpital de la Croix-Rousse, Hospices Civils de Lyon. These strains were selected for their specific antibiotic susceptibilities regarding gentamicin or vancomycin or clindamycin. Resistance profiles were determined with Vitek 2 (Biomérieux) by the routine Bacteriology laboratory. All the strains were tested with Crystal Violet method beforehand to ensure that they can form at least moderate biofilm regarding Stepanovic's classification (19). The strains are presented in Table 1.

**Table 1:** List of the bacterial strains used in this study and their antibiotic resistance profiles.

	**Cefotaxime**	**Gentamicin**	**Vancomycin**	**Clindamycin**
MSSA	S	S	S	S
MRSA	**R**	S	S	S
GentaR MRSA	**R**	**R**	S	S
VancoR MSSA	S	S	**R**	S
ClindaR MSSA	S	S	S	**R**
MSSE	S	S	S	S
MRSE	**R**	S	S	S
GentaR MSSE	S	**R**	S	S
MRSE VancoR	**R**	S	**R**	S
ClindaR MRSE	**R**	S	S	**R**

*Resistance profiles were determined with Vitek 2 in the routine laboratory. R, resistant; S, susceptible*.

### Antibiotic-Loaded Bone Cements

Four bone cements commercialized by Heraeus Medical were used in this study: plain cement (without antibiotic), cement loaded with gentamicin alone (G), cement loaded with gentamicin plus vancomycin (G + V), and cement loaded with gentamicin plus clindamycin (G + C). Disk-like specimens (diameter 2.5 cm, height 1.0 cm) were used. Specific antibiotic loads for each cement are presented in Table 2.

**Table 2:** List of the bone cements used in this study and their characteristics.

**Cement**	**Antibiotic and quantity**				**Commercial name**
Plain	–	–	–	–	PALACOS R
G	Gentamicin	0.5 g	–	–	PALACOS R + G
G + V	Gentamicin	0.5 g	Vancomycin	2 g	COPAL G + V
G + C	Gentamicin	1 g	Clindamycin	1 g	COPAL G + C

### Preparation of ALBC Elution Solutions

**Figure 1:**
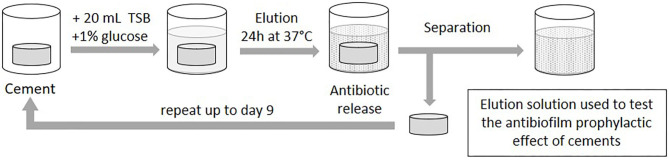
Preparation of elution solutions. A specimen of cement was placed in 20 mL of TSB supplemented with 1% of glucose and incubated at 37°C. Each day, the media were discarded, and new media were added to the cements. Elution solutions for Day 1, Day 3, and Day 9 were used for biofilm experiments.

To evaluate the effect of ALBCs against biofilm formation, we prepared elution solutions that contain antibiotics released from ALBCs. Disk-like specimens were incubated in 20 mL of Tryptic Soy Broth (TSB, Bacto) supplemented with 1% of glucose (an artificial medium that favors strong biofilm formation in 24 h) in Falcon tube 25 mL. The ALBCs were incubated for 1–9 days at 37°C (Figure 1). Indeed, most of prosthesis inoculation occurs during the surgery or during the few days after the surgery, as the scar and the joint cavity are not yet impervious. Consequently, eluted antibiotics that have prolonged effect to limit the formation of the biofilm could be of importance to prevent the bacterial inoculation immediately after the surgery. The media were changed daily. For the biofilm formation experiments, ALBC elution solutions from Day 1, Day 3, and Day 9 were used.

### Disk Diffusion Assay With ALBC Elution Solutions

Bacterial suspensions were prepared in saline solution and adjusted at 0.5 McFarland for each strain. Then, the bacterial suspensions were swabbed on Muller Hinton agar plates over the entire agar surface. After inoculation, sterile disks with a diameter of 6 mm were applied on the inoculated plates. Disks were impregnated with 20 μL of each ALBC elution solution. Plates were incubated for 24 h before the measurement of the diameters of inhibition zones. Two independent experiments were performed in technical duplicate.

### Determination of the Prophylactic Anti-biofilm Effect of ALBC Elution Solutions

Overnight cultures of *S. aureus* or *S. epidermidis* in liquid Brain Heart Infusion (BHI) were standardized to OD600 = 1 ± 0.05 before being diluted at 1:100 in ALBC elution solutions (Day 1, Day 3, and Day 9), and 100 μL was added in a 96-well plate (Greiner Bio-One) for 24 h of incubation at 37°C in humid atmosphere. After 24 h, the supernatant was removed, and biofilms were washed for 45 min using Biofilm Care (20). This smooth washing method favors the preservation of the biofilm that otherwise can be denatured with classic washing methods. Biofilms were then resuspended in 200 μL of phosphate buffer saline (PBS) by scraping the wells using sterile pipette tips and sonicating for 10 min at 40 Hz using Bactosonic (Bandelin). Finally, the number of viable bacteria inside the biofilm was evaluated with plate counting on COS agar plates (Biomérieux).

### Graphical Representation and Statistical Analysis

For each condition, three independent experiments in technical experiments (three wells for each condition for each experiment) were performed. Results were presented as inhibition of biofilm formation by comparing G, G + V, and G + C data to plain cement (no antibiotic) data. Data (nine values per condition) were presented as histograms (median with range). Due to the number of values, non-parametric statistical analysis was performed. We performed Kruskal–Wallis tests comparing the data at each day. Then, we performed first tests to compare G, G + V, and G + C to the control condition (plain cement). Then, we performed second Dunn's multiple comparisons to compare G, G + V, and G + C with each other. All analyses were performed using Prism software (GraphPad, San Diego, CA, USA).

## Results

### Effects of ALBC Elution Solutions Against Planktonic Bacteria

We first performed disk diffusion assays with the ALBC elution solutions to observe the effect of ALBCs against planktonic staphylococci (Table 3). For plain cements, the diameters were 6 mm, which is the diameter of the disk. It means that there was no antibacterial effect of the elution solutions from plain cement. At Day 1, we observed that almost all the ALBCs had an antibacterial effect. The only exceptions were for the gentamicin-resistant MRSA and methicillin-susceptible *Staphylococcus epidermidis* (MSSE) and the vancomycin-resistant when they are exposed to G and G + C cements. At Days 3 and 9, a decrease of antibacterial activity is observed for all ALBCs. Only G + C kept an antibacterial activity against all the strains except the clindamycin-resistant MSSA.

**Table 3:** Disk diffusion assay with ALBC elution solutions.

	**Day 1**				**Day 3**				**Day 9**			
	**Plain**	**G**	**G + V**	**G + C**	**Plain**	**G**	**G + V**	**G + C**	**Plain**	**G**	**G + V**	**G + C**
MSSA	6 ± 0	14.5 ± 0.7	14 ± 0	27.5 ± 3.5	6 ± 0	6.5 ± 0.7	7.5 ± 0.7	21.5 ± 2.1	6 ± 0	6 ± 0	6 ± 0	21 ± 1.4
MRSA	6 ± 0	13 ± 2.8	14.5 ± 3.5	26 ± 1.4	6 ± 0	8 ± 1.4	8.5 ± 0.7	21.5 ± 0.7	6 ± 0	6 ± 0	6 ± 0	20 ± 1.4
GentaR MRSA	6 ± 0	6 ± 0	7.5 ± 0.7	25.5 ± 2.1	6 ± 0	6 ± 0	6 ± 0	20 ± 2.8	6 ± 0	6 ± 0	6 ± 0	19 ± 1.4
VancoR MSSA	6 ± 0	15.5 ± 0.7	14.5 ± 0.7	25.5 ± 0.7	6 ± 0	6.5 ± 0.7	7 ± 0	19 ± 1.4	6 ± 0	6 ± 0	6 ± 0	17.5 ± 0.7
ClindaR MSSA	6 ± 0	11.5 ± 2.1	12.5 ± 3.5	16 ± 1.4	6 ± 0	6 ± 0	7 ± 1.4	8.5 ± 2.1	6 ± 0	6 ± 0	6 ± 0	8 ± 1.4
MSSE	6 ± 0	21 ± 1.4	18.5 ± 0.7	25.5 ± 2.1	6 ± 0	10.5 ± 0.7	11 ± 1.4	20 ± 2.8	6 ± 0	6.5 ± 0.7	6.5 ± 0.7	19.5 ± 0.7
MRSE	6 ± 0	23 ± 4.2	23 ± 4.2	27 ± 4.2	6 ± 0	14 ± 1.4	15.5 ± 0.7	20 ± 2.8	6 ± 0	11 ± 1.4	10.5 ± 0.7	18.5 ± 2.1
GentaR MSSE	6 ± 0	6 ± 0	6 ± 0	27.5 ± 3.5	6 ± 0	6 ± 0	6 ± 0	20.5 ± 3.5	6 ± 0	6 ± 0	6 ± 0	18.5 ± 0.7
VancoR MRSE	6 ± 0	19 ± 1.4	19 ± 1.4	26 ± 2.8	6 ± 0	9.5 ± 0.7	10 ± 0	19 ± 1.4	6 ± 0	6 ± 0	6 ± 0	17 ± 0
ClindaR MRSE	6 ± 0	22 ± 2.8	20.5 ± 3.5	26 ± 2.8	6 ± 0	11.5 ± 0.7	13 ± 1.4	19 ± 1.4	6 ± 0	7 ± 1.4	7 ± 1.4	16.5 ± 0.7
Mean	6.0	15.2	15.0	25.3	6.0	8.5	9.15	18.9	6.0	6.7	6.6	17.6
SD	0.0	6.2	5.5	3.3	0.0	2.8	3.2	3.8	0.0	1.6	1.4	3.6

*Results are presented as mean ± SD. Values are in mm*.

### Inhibition of Biofilm Formation by ALBCs With Multi-Susceptible or Only Methicillin-Resistant Staphylococcal Strains

**Figure 2:**
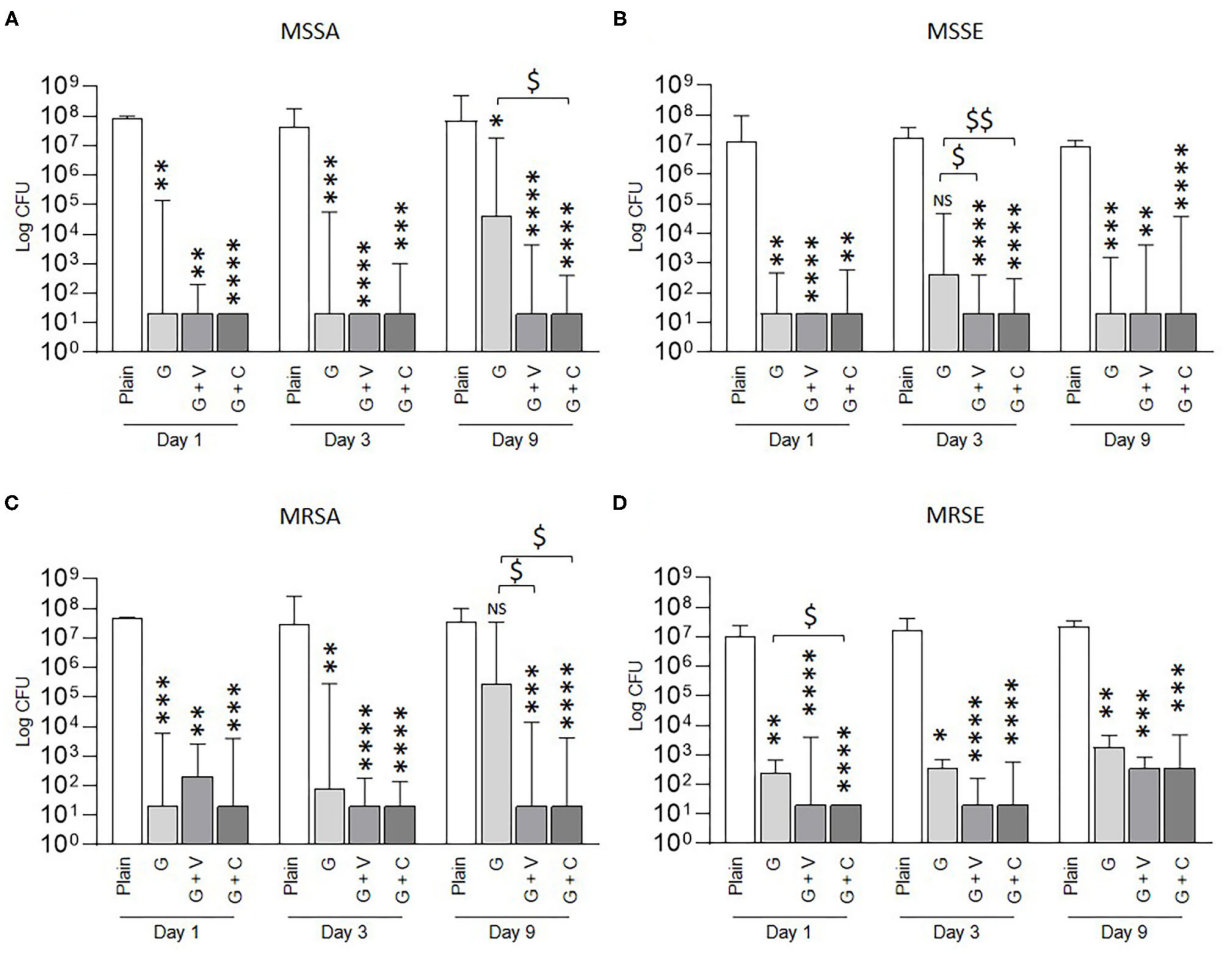
Prophylactic anti-biofilm effect of ALBCs against MSSA strain **(A)**, MSSE strain **(B)**, MRSA strain **(C)**, and MRSE strain **(D)**. Three independent experiments in technical experiments (three wells for each condition for each experiment) were performed. Non-parametric Kruskal–Wallis tests were performed to compare the data at each day. A Dunn's multiple comparisons test was performed as follow-up test. For each day, *, **, ***, and **** mean *p* < 0.05, *p* < 0.01, *p* < 0.001, and *p* < 0.0001, respectively, in comparison with plain cement (control without antibiotic). For each day, $ and $$ mean *p* < 0.05, *p* < 0.01, respectively, in comparison with G cement.

Then, we investigated the prophylactic anti-biofilm effect of ALBCs against methicillin-susceptible strains (Figures 2A,B). After 24 h of incubation in the elution solutions, 10^8^ and 10^7^ CFU were counted for plain cement for MSSA and MSSE, respectively. For both strains, all ALBCs (G, G + V, and G + C) decreased the biofilm formation without difference whatever the time of elution (Day 1, Day 3, or Day 9). Median values of 10^1^ CFU were reported for all the three ALBCs. However, two exceptions were present: at Day 9 for MSSA, we observed an increase of CFU counts to 10^4^ for G and a significant difference between the efficacy of G + C and G (Figure 2A); at Day 3 for MSSE, the number of CFU increased over 10^2^ for G, and significant differences were observed between G + V and G + C when compared with G (Figure 2B).

Similar results were obtained when the methicillin-resistant strains [MRSA and methicillin-resistant *Staphylococcus epidermidis* (MRSE)] were tested, with globally no significant difference between the biofilm inhibition effect of G, G + V, and G + C (Figures 2C,D). Again, we reported two exceptions: at Day 9 for MRSA, the CFU count increases to 10^5^ and was not significantly different from G. Moreover, the CFU counts for G + V and G + C were statistically different from G (Figure 2C). The other exception was a significant difference between G and G + C for the MRSE strain at Day 1 that was not reproduced at Day 3 or Day 9 (Figure 2D).

G, G + V, and G + C cements shared the same ability to inhibit biofilm formation from MSSA, MSSE, MRSA, and MRSE strains with some exceptions that are strain and time dependent.

### Inhibition of Biofilm Formation by ALBCs With Gentamicin-Resistant Staphylococcal Strains

**Figure 3:**
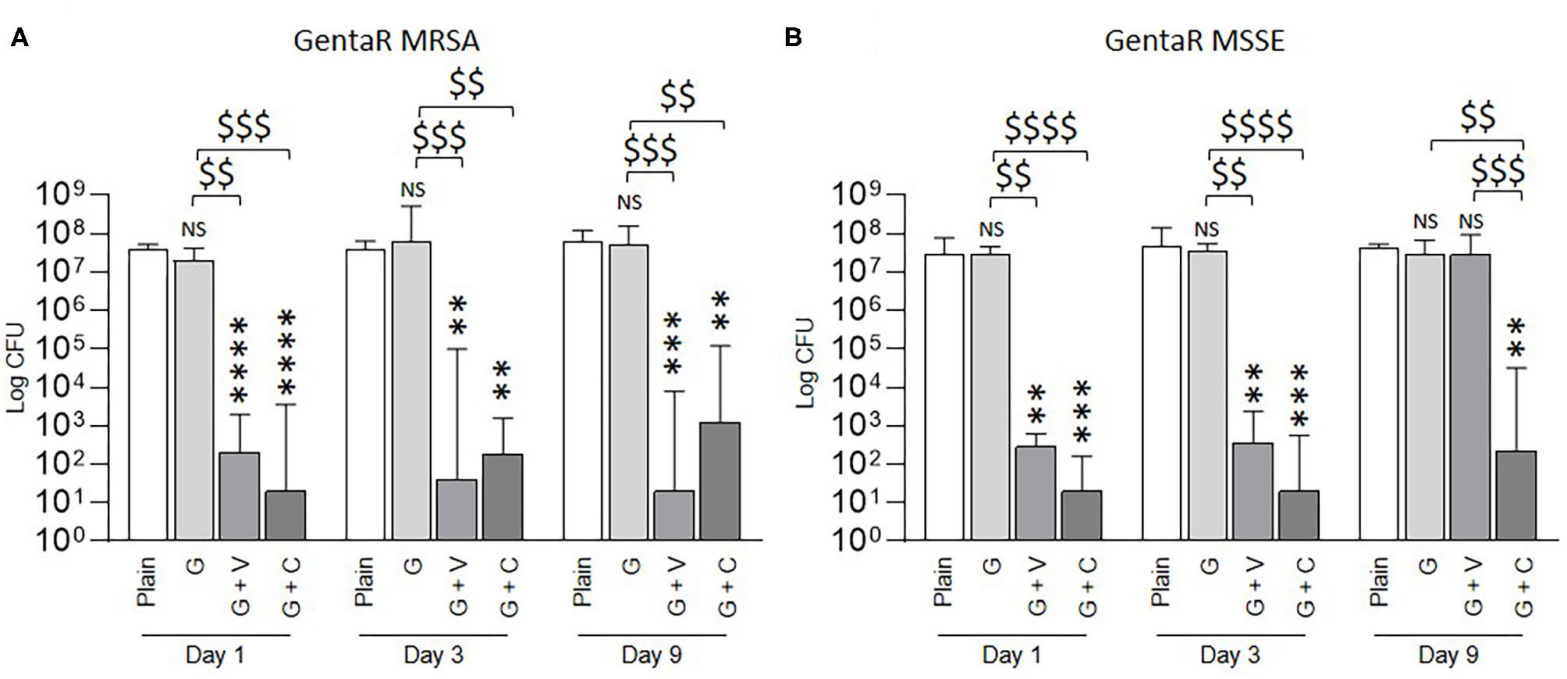
Prophylactic anti-biofilm effect of ALBCs against gentamicin-resistant MRSA **(A)** strain and gentamicin-resistant MSSE strain **(B)**. Three independent experiments in technical experiments (three wells for each condition for each experiment) were performed. Non-parametric Kruskal–Wallis tests were performed to compare the data at each day. A Dunn's multiple comparisons test was performed as follow-up test. For each day, **, ***, and **** above the plot mean *p* < 0.01, *p* < 0.001, and *p* < 0.0001, respectively, in comparison with plain cement (control without antibiotic). For each day, $$, $$$, and $$$$ mean *p* < 0.01, *p* < 0.001, and *p* < 0.0001, respectively, in comparison with G cement.

We then tested the ability of ALBCs to inhibit the formation of biofilm by gentamicin-resistant staphylococcal strains (Figures 3A,B). With elution solution from plain cement, the bacterial count was between 10^7^ and 10^8^ CFU. The G cement did not permit to inhibit the formation of biofilm by the gentamicin-resistant strains (Figures 3A,B) whatever the elution solutions used (Day 1, Day 3, or Day 9). The results for G were like the ones obtained with the plain cement. For G + V and G + C, CFU count was between 10^1^ and 10^3^, corresponding to at least a 4-Log decrease. An exception was observed at Day 9 for the gentamicin-resistant MSSE where G + C kept its ability to significantly decrease biofilm formation, whereas G + V had no effect on biofilm formation (Figure 3B).

G + V and G + C had a significant better ability to inhibit biofilm formation than G cement for the two gentamicin-resistant tested strains.

### Inhibition of Biofilm Formation by ALBCs With Vancomycin-Resistant or Clindamycin-Resistant Staphylococcal Strains

**Figure 4:**
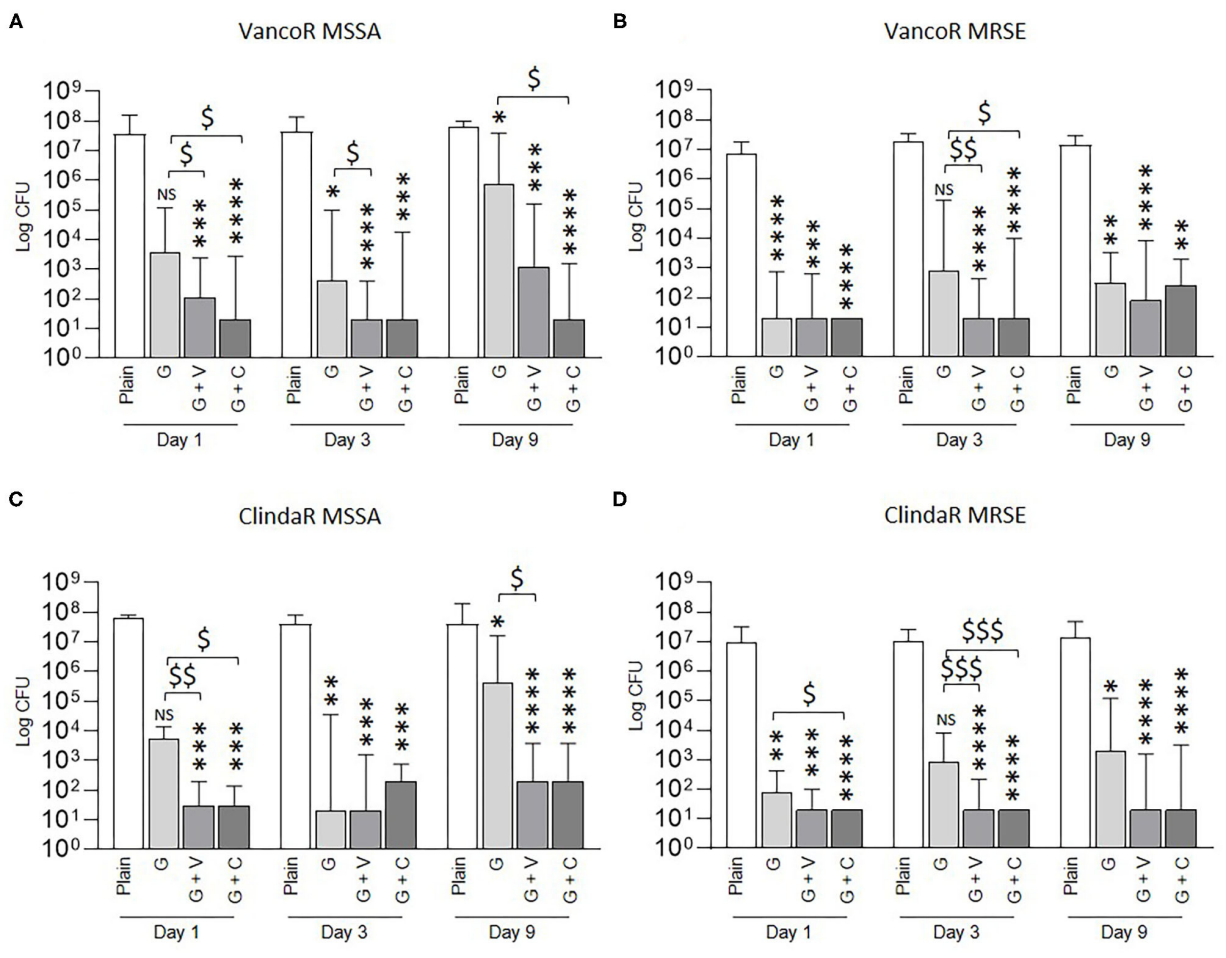
Prophylactic anti-biofilm effect of ALBCs against vancomycin-resistant MSSA strain **(A)**, vancomycin-resistant MRSE strain **(B)**, clindamycin-resistant MSSA strain **(C)**, and clindamycin-MRSE strain **(D)**. Three independent experiments in technical experiments (three wells for each condition for each experiment) were performed. Non-parametric Kruskal–Wallis tests were performed to compare the data at each day. A Dunn's multiple comparisons test was performed as follow-up test. For each day, *, **, ***, and **** above the plot mean *p* < 0.05, *p* < 0.01, *p* < 0.001, and *p* < 0.0001, respectively, in comparison with plain cement (control without antibiotic). For each day, $, $$, and $$$ mean *p* < 0.05, *p* < 0.01, and *p* < 0.001, respectively, in comparison with G cement.

We next tested the efficacy of ALBCs to inhibit biofilm formation by vancomycin- and clindamycin-resistant staphylococcal strains (Figure 4). Globally, all the ALBCs were able to significantly decrease biofilm formation for the four tested strains. As seen in Figure 1 for the MSSA, MSSE, MRSA, and MRSE strains, we observed strain- and time-dependent exceptions. Indeed, the effect of G cement was not significant for the vancomycin-resistant and the clindamycin-resistant MSSA strains at Day 1 (Figures 4A,C). The same observations were made at Day 3 for the vancomycin-resistant and clindamycin-resistant MRSE strains (Figures 4B,D). In both cases, the CFU counts of G + V and G + C were significantly lower than that of G cement. In other specific situations, we observed significant differences between G + V or G + C and G (Figures 4A,C,D).

As observed in Figure 1, G, G + V, and G + C cements shared the same ability to inhibit biofilm formation from vancomycin-resistant and clindamycin-resistant staphylococci with some exceptions that are strain and time dependent.

### Global Analysis With Pooled Results for *S. aureus* and *S. epidermidis* Strains

**Figure 5:**
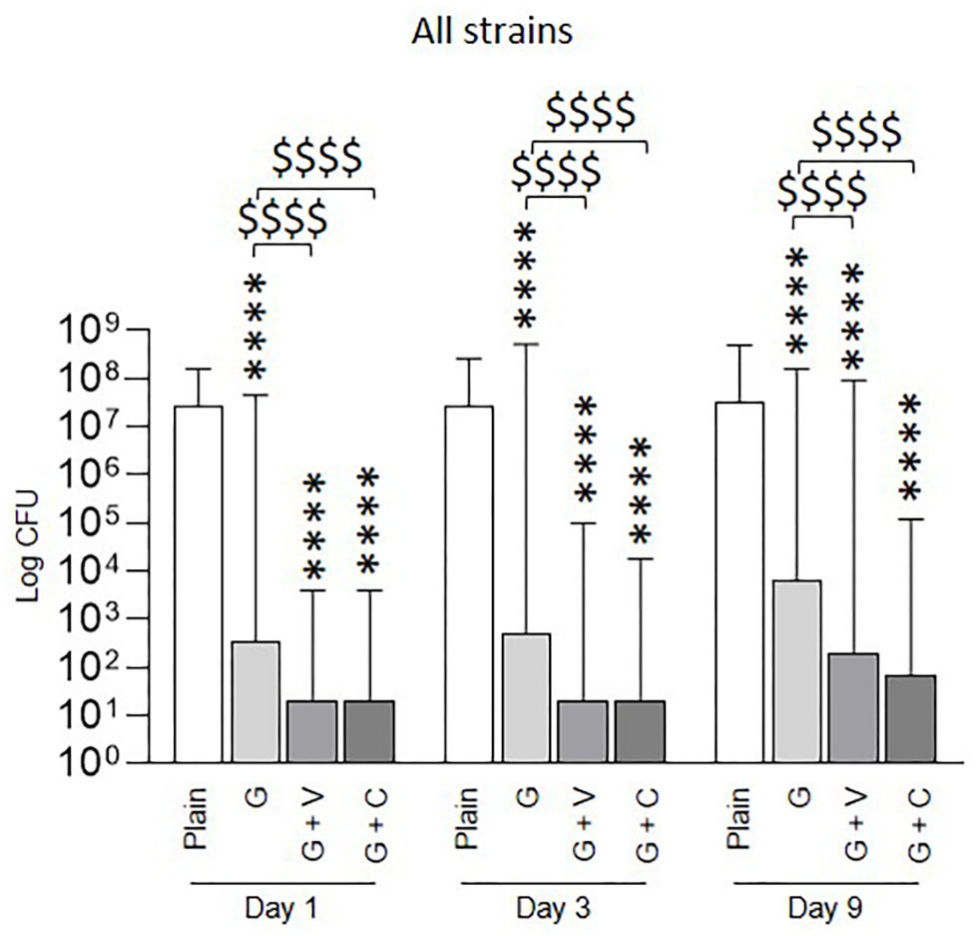
Prophylactic anti-biofilm effect of ALBCs against all the tested staphylococcal strains (10 strains pooled). Three independent experiments in technical experiments (three wells for each condition for each experiment) were performed. Non-parametric Kruskal–Wallis tests were performed to compare the data at each day. A Dunn's multiple comparisons test was performed as follow-up test. For each day, **** above the plot means *p* < 0.0001 in comparison with plain cement (control without antibiotic). For each day, $$$$ means *p* < 0.0001, respectively, in comparison with G cement.

To have an overview of the anti-biofilm activity of ALBCs, the results from the 10 staphylococcal strains were pooled in one graph (Figure 5). At each day, all ALBCs were significantly able to decrease biofilm formation when compared with plain cement. Moreover, G + V had a significantly better anti-biofilm effect than G cement on gentamicin-resistant staphylococci that represent 20% of the total bacterial population.

## Discussion

The use of ALBC for prosthesis fixation in primary total arthroplasties or revision surgeries is still debated. Indeed, various studies reported opposite results. However, in these studies, the type of ALBC (handmade or commercial) and the type of antibiotics loaded in the cement (gentamicin alone, gentamicin coupled with another antibiotic) are rarely taken in consideration. In this study, we investigated the *in vitro* anti-biofilm activity of commercially available ALBCs with low doses of antibiotics. Using low-dosed ALBCs is primordial to minimize the negative effects on cement mechanical properties that can be observed with high-dosed ALBCs. However, it was reported that low-dosed ALBCs can favor the induction of resistance, especially when gentamicin alone is used (21).

In this study, we chose to use elution solutions to investigate the effect of ALBCs on biofilm formation. Previous studies mostly focused on biofilm formation directly on the cement, investigating the biofilm formation by microscopy or by direct interaction between cement and bacterial culture on agar plate (22). By studying the effect of elution solutions on biofilm formation on an independent material (in our case, the bottom of 96-well plates), we placed ourselves in the context that ALBCs have to prevent biofilm formation on themselves but also on the prosthesis (metallic or polyethylene components) or on bone or soft tissues. Moreover, we chose to use the classical conditions of biofilm formation, using a rich nutrient medium supplemented with glucose and a high inoculum, both favoring a rapid and intense development of biofilm. These conditions are not the best to easily prove the anti-biofilm effect of ALBCs and can explain why in most of our experiments, we did not reach a total inhibition of biofilm formation. Finally, the elution solutions were changed every day to mimic the depletion/elimination of antibiotics that happens in the joint. It means that the concentrations of antibiotics are lower at Day 9 than at Day 1. However, our conditions can be considered as not clinically relevant. Indeed, the formation of biofilm takes several days in patients, and synovial fluid and bone environment can be considered as poor media for biofilm formation. The time and the type of media for biofilm formation could influence the biofilm structure and composition and then impact the susceptibility to antibiotics. Regarding our method, we chose to use CFU counting. This method is the standard for bacterial counting but suffers from low reproducibility. Confocal microscopy is the method of choice for imaging and determining biofilm formation, but as we tested 120 conditions (10 strains, four cements, and three conditions), CFU counting seemed more accurate.

Synergistic activities of gentamicin plus vancomycin or gentamicin plus clindamycin against staphylococci have been known for almost 40 years (23–25). Ensing et al. observed a higher effect of the G + C cement (COPAL G + C) than of the G cement (PALACOS R + G) (22). In their article, they observed that the antibiotic release from the G + C cement was more important than the one from the G cement, explaining the higher activity of G + C. The authors also reported that the gentamicin-susceptible *S. aureus* that they used for their study formed gentamicin-resistant small colony variants (SCVs) on the G cement (22). However, COPAL G + C contains more gentamicin (1 g) than PALACOS R + G (0.5 g). This difference could also explain the higher activity of G + C. In our study, we observed a difference of biofilm inhibition between G + V or G + C and G cement when we tested the gentamicin-resistant staphylococci. In the global population of staphylococci, *S. aureus* and CNS, such as *S. epidermidis*, the prevalence for gentamicin resistance can vary between 23 and 79% (17, 18). It means that staphylococcal PJIs have a non-negligible possibility to be caused by a gentamicin-resistant strain. In this context, using an ALBC combining gentamicin to another antibiotic appears warranted. In our study, we tested a gentamicin-resistant MRSA strain and a gentamicin-resistant MSSE strain. In both cases, gentamicin alone cannot prevent biofilm formation, even after Day 1 of elution, when the concentration of antibiotic is the most elevated. Indeed, even with a concentration of gentamicin around 100 μg/mL as it can be dosed at Day 1 (data not shown), the concentration is too low to overpass gentamicin resistance mechanisms and to prevent biofilm. However, for ALBCs loaded with gentamicin coupled with vancomycin or clindamycin, biofilm formation was prevented, even when elution solutions from Day 9 were used (Figure 3).

Regarding these previous results, it could be tempting to wonder that ALBCs with only vancomycin or clindamycin would be enough for preventing PJIs. However, PJIs are not only due to staphylococci, and vancomycin or clindamycin is not efficient against Gram-negative bacteria. Moreover, staphylococci can be resistant to vancomycin, even if it is not frequent, or to clindamycin, which concerned 79% of *S. epidermidis* strains in the study by Hellmark et al. (18). In our study, we tested two vancomycin-resistant strains (MSSA and MRSE) and two clindamycin-resistant strains (MSSA and MRSE). For these four strains, all ALBCs were able to prevent biofilm formation (Figure 4). However, anti-biofilm activity appears more pronounced for G + V and G + C than for gentamicin alone after 9 days of elution for clindamycin-resistant strains (Figure 4). For COPAL G + V cements, the higher anti-biofilm effect could be logically attributed to the presence of vancomycin. For the COPAL G + C cements, the higher dose of gentamicin could allow a better inhibition of biofilm formation than that of cement with gentamicin alone (Figure 4, Table 2). Our results highlight that the combination of antibiotics can potentialize the anti-biofilm effect even if the strain is resistant to one of the loaded antibiotics. Moreover, it is important to take into account that the chemicophysical properties of ALBCs differ between the G cement and the G + V and G + C cements. Indeed, PALACOS R + G and COPAL G + V and COPAL G + C have different porosities that impact the release of the antibiotics and can potentially explain the higher effect of G + V and G + C in specific conditions.

Regarding the mechanisms involved in the inhibition of biofilm, two mechanisms are identified: (i) killing the planktonic staphylococci that will not form biofilm thereafter and/or (ii) acting directly against adhering staphylococci during the early step of biofilm formation. In our study, the staphylococci are directly exposed to antibiotics as they grow in ALBC elution solutions, so we cannot differentiate which mechanism is involved. It seems logical that the antibiotics present in the solution first attack the planktonic bacteria, and that the decrease of activity sometimes observed at Day 9 with the G cement is due to a lower concentration in antibiotics. However, when we compared our results between the disk diffusion assays and the biofilm inhibition assays, we observed difference regarding the activity. Indeed, even though G and G + V lost their activity in disk diffusion assay (Table 3), they kept a good activity against biofilm formation (Figures 2–5). G and G + V cements can inhibit biofilm formation at concentrations that are not sufficient to inhibit bacterial growth in disk diffusion assay. These results suggest that G and G + V cements have a specific activity against biofilm formation that is different from the bactericidal/bacteriostatic activity. Their activities not only are due to the killing of planktonic staphylococci before biofilm formation but also involve a specific effect against biofilm formation. Regarding G + C cement, we observed an antibacterial effect until Day 9 in disk diffusion. In this case, we can hypothesize that the killing of planktonic bacteria before biofilm formation has a more important role than with G and G + V cements in the global activity against biofilm formation.

Finally, concerning clinical evidence, recent meta-analysis did not highlight differences in PJI rates between primary plain-cemented and ALBC-cemented TJA and point out differences between primary TKA and primary THA. However, as the parameters of ALBCs (commercially available or handmade; only gentamicin or gentamicin with another antibiotic) were not taken into account, it appears essential to investigate the impact of using commercial ALBCs following cemented TJA and the impact of adding vancomycin or clindamycin in clinical trials.

## Conclusion

Our *in vitro* results suggest that using commercially available ALBCs loaded with gentamicin added with vancomycin or clindamycin for prosthesis fixation can help in preventing staphylococcal PJIs following primary TJA, non-septic TJA revisions or septic TJA revisions, especially PJIs caused by gentamicin-resistant staphylococci. Moreover, our results suggest that elution solutions from ALBCs can prevent biofilm formation at concentrations that are not able to inhibit bacterial growth in disk diffusion assays.

## Data Availability Statement

The raw data supporting the conclusions of this article will be made available by the authors, without undue reservation.

## Author Contributions

AC, MB, and CH performed the experiments and analyzed the results. JJ defined the study plan, analyzed the results, and drafted the manuscript with TF and FL. All authors reviewed and accepted the manuscript.

## Conflict of Interest

The authors declare that the research was conducted in the absence of any commercial or financial relationships that could be constructed as a potential conflict of interest.
